# Genome-Wide Association Study Reveals a New QTL for Salinity Tolerance in Barley (*Hordeum vulgare* L.)

**DOI:** 10.3389/fpls.2016.00946

**Published:** 2016-06-28

**Authors:** Yun Fan, Gaofeng Zhou, Sergey Shabala, Zhong-Hua Chen, Shengguan Cai, Chengdao Li, Meixue Zhou

**Affiliations:** ^1^School of Land and Food and Tasmanian Institute for Agriculture, University of Tasmania,Kings Meadows, TAS Australia; ^2^Western Australian State Agricultural Biotechnology Centre, Murdoch University,Murdoch, WA Australia; ^3^School of Science and Health, Western Sydney University,Penrith, NSW Australia

**Keywords:** genome wide association study, QTL mapping, evaluation methods, salinity tolerance, barley

## Abstract

Salinity stress is one of the most severe abiotic stresses that affect agricultural production. Genome wide association study (GWAS) has been widely used to detect genetic variations in extensive natural accessions with more recombination and higher resolution. In this study, 206 barley accessions collected worldwide were genotyped with 408 Diversity Arrays Technology (DArT) markers and evaluated for salinity stress tolerance using salinity tolerance score – a reliable trait developed in our previous work. GWAS for salinity tolerance had been conducted through a general linkage model and a mixed linkage model based on population structure and kinship. A total of 24 significant marker-trait associations were identified. A QTL on 4H with the nearest marker of bPb-9668 was consistently detected in all different methods. This QTL has not been reported before and is worth to be further confirmed with bi-parental populations.

## Introduction

Salinity stress disrupts plant metabolisms, affecting crop yield and restricting the utilization of agricultural land. It has been estimated that 20% of arable land worldwide is salinized which mainly results from natural causes, such as climate change and human influence factors like poor irrigation management (Flowers and Yeo, 1995; FAO, 2008). At the whole-plant level, salinity stress is considered to be composed of two phases: a rapid osmotic stress which reduces shoot growth, and slower ionic stress which accelerates senescence of older leaves due to elevated leaf Na^+^ content (Munns and Tester, 2008). Osmotic stress affects plant growth by reducing cell expansion and elongation rates, which leads to smaller and thicker leaves, and down-regulated photosynthesis by reducing stomatal aperture (Bradford, 1976). Plants employ numerous mechanisms to adapt to saline conditions. The major ones include Na^+^ exclusion from uptake; control of xylem Na^+^ loading and/or its retrieval from the shoot; efficient vacuolar Na^+^ sequestration; cytosolic K^+^ homeostasis and retention in root and mesophyll cells; efficient osmotic adjustment; and ROS detoxification (Zhu, 2003; Munns and Tester, 2008). Some naturally salt tolerant species such as halophytes also possess a set of unique anatomical features such as salt grands of bladders (Flowers and Colmer, 2008; Shabala and Mackay, 2011; Shabala et al., 2014). Ion homeostasis is controlled by numerous ion channels, ion sensing and signaling, pathways of transportation and compartmentalization mechanisms (Zhu, 2003; Munns and Tester, 2008). Since many traits underlying adaption to stress are quantitative and controlled by multiple genetic pathways, a wide variety of genes are implicated in salinity tolerance (DeRose-Wilson and Gaut, 2011).

Barley is one of the most important cereal crops worldwide, and also the most salt tolerant cereal (Munns and Tester, 2008). Cultivated barley originated from wild barley and domesticated within the Fertile Crescent and Tibet (Badr et al., 2000; Kilian et al., 2006; Dai et al., 2012). Barley is indispensable to malting and brewing industries and also serves as a staple food in some areas of the world due to its broad adaption to salinity, drought, and high altitude (Baik and Ullrich, 2008). Both genetic diversity and adaption to broad conditions resulted in a rich gene pool of barley (Nevo and Chen, 2010). However, modern cultivated barley varieties only include 15 to 40% of all alleles within the barley gene pool, indicating that only a small part of barley genetic potential has been used for improvement for salinity tolerance (Ellis et al., 2000; Kilian et al., 2006; Long et al., 2013). Progress in improving crop salinity tolerance or developing salt tolerant cultivars has been lagging behind many improvements in crop biotic stress tolerance due to the fact that salinity tolerance is a physiologically and genetically (quantitative inheritance) complex trait controlled by numerous QTL (Flowers, 2004). Traditional bi-parental QTL mapping has been widely used for the dissection of salinity tolerance and the identification of tolerance genes. Bi-parental QTL mapping detects chromosomal regions varying from a few to several tens of centi-Morgans (cM), harboring a large number of genes (Long et al., 2013). Many QTL for salinity tolerance were detected using a wide variety of agronomic and physiological traits as selection criteria for barley salinity tolerance. These include plant survival (Xu et al., 2012; Zhou et al., 2012; Fan et al., 2015), yield and agronomic traits (Ellis et al., 2002; Xue et al., 2009), seed germination (Witzel et al., 2010), Na^+^ exclusion (Shavrukov et al., 2010), tissue ion content (Xue et al., 2009), water soluble carbohydrate and chlorophyll content (Siahsar and Narouei, 2010).

Bi-parental QTL mapping has shown the power to identify candidate QTL/genes for salinity tolerance. However, allelic diversity between parents and recombination occurring during the production of populations are limited, which leads to limitations in QTL mapping, although there are some multi-parent populations such as Multi-parent Advanced Generation Inter-Cross (MAGIC) (Kover et al., 2009; Korte and Farlow, 2013). Recent rapid development in genotyping and sequencing technologies has enabled novel association mapping to identify alleles in a much broader range of natural accessions. A genome wide association study (GWAS) explores the recombination that has occurred during a long evolutionary history of diverse sets of accessions (Nordborg and Tavare, 2002). QTL mapping is suitable for detecting rare alleles of large effect, while GWAS could be a complementary approach for the identification of major allelic variants underlying quantitative and complex traits (DeRose-Wilson and Gaut, 2011; Long et al., 2013). Barley has a high level population structure such as two-rowed and six-rowed cultivars, spring and winter barley (Pasam et al., 2012). Due to the confounding effect of population structure, GWAS have a higher chances of producing false positive (type I) and negative (type II) errors than QTL mapping (Zhu et al., 2008). A mixed-linear model (MLM) approach has been developed which leads to a better performance (Yu et al., 2006). In barley, GWAS has been used for detecting genetic variations underlying diverse complex traits such as agronomic and morphologic traits (Cockram et al., 2010; Pasam et al., 2012; Wang et al., 2012; Munoz-Amatriain et al., 2014), malting quality related traits (Huang et al., 2014; Matthies et al., 2014; Cai et al., 2015), cadmium accumulation (Wu et al., 2015), frost tolerance (Visioni et al., 2013), aluminum tolerance (Cai et al., 2013; Zhou et al., 2016) and salinity tolerance (Long et al., 2013).

The objectives of this study were to (1) detect candidate QTL for salinity tolerance in barley through a GWAS; (2) and discuss how statistical models affect the power of GWAS. Also, for the first time, we utilized QTL mapping through MapQTL 6.0 software to confirm those QTL detected in GWAS.

## Materials and Methods

### Barley Germplasm and Genotyping

A total of 206 barley accessions collected from Europe, Asia, Australia, and Canada were used in this study. All the accessions were genotyped with Diversity Arrays Technology (DArT) markers (Wenzl et al., 2004) and distributed over the whole genome. A consensus genetic map was sourced from http://www.diversityarrays.com. More than 1100 polymorphic DArT markers were scored for this population. Among them, 482 markers were found to have a specific chromosome position. A total of 408 markers, with *Q* value (marker quality) and call rate above 80% as well as minor allele frequency (MAF) higher than 0.05, were used for population structure and association mapping analysis.

### Evaluation of Salinity Tolerance

Salinity tolerance of these barley varieties were evaluated in the 2013 and 2014 barley growing seasons. Experiments were conducted in a glasshouse in Launceston, Tasmania, Australia. Seeds of all the accessions were sown in large plastic containers (1.6 m × 2.5 m × 0.6 m) using a potting mixture described in Fan et al. (2015). Each genotype consisted of three replicates, each of five seedlings. Salt treatment was performed with 300 mM NaCl. A control experiment was not conducted since it had been proved that different varieties, in the same potting mixture without salt added, exhibited no obvious symptoms of leaf chlorosis or wilting (Zhou et al., 2012). The salt treatment started at the two-leaf stage and was repeated every 3 days according to our previous method (Xu et al., 2012; Zhou et al., 2012). When the most susceptible lines showed severe symptoms, salinity tolerance was assessed by combining scores for plant survival and leaf chlorosis (0 = no damage and 10 = all dead) (Xu et al., 2012).

### Population Structure and Kinship Analysis

A total of 408 DArT markers distributed over the whole genome were used for population structure analysis using STRUCTURE software (v2.3.3) (Pritchard et al., 2000). The number of clusters (K) was set from 2 to 12 and 20 iterations were conducted in an admixture model with a 10,000 burning period and 10,000 MCMC (Markov Chain Monte Carlo). *K* value was the number of clusters when ΔK achieved maximum value (Evanno et al., 2005). Principle component analysis (PCA) was performed using GAPIT R package to visualize the dispersion of the association panel in a graph (Lipka et al., 2012). A kinship analysis was conducted using SPAGeDi software (Hardy and Vekemans, 2002). The kinship matrix measured the genetic similarity between individuals.

### Genome Wide Association Study

A GWAS among phenotypic trait (mean value of 2013 and 2014), DArT markers (genotype), population structure and kinship were conducted using TASSEL software (v3.0) (Bradbury et al., 2007). The Q, K and Q + K methods were used for GWAS. For Q model: *y* = Xβ + Qν + e; for K model: *y* = Xβ + Zμ + e; for Q + K model: *y* = Xβ + Qν + Zμ + e. X is DArT marker matrix, Q and Z represent sub-population membership matrix and kinship matrix, respectively, β and ν are coefficient vectors for DArT marker and sub-population membership, respectively, μ is a vector of random genetic effects μ ~ N (0, 2 K) and e is the random error vector. *P* < 0.01 (-log_10_ (P) > 2) was set as the significant threshold in the association study. Manhattan plots were displayed using R software (v2.14.2). For evaluating the fitness and efficiency of different models, quantile–quantile (Q–Q) plots were shown using TASSEL (v3.0).

### Confirmation of the Number of QTL

A genetic linkage map for this natural population has been constructed using Diversity Array Technology (DArT) markers. The DArT markers consensus genetic map was provided at http://www.diversityarrays.com. The software package MapQTL 6.0 (Van Ooijen, 2009) was also used to detect QTL and confirm the relationship between different markers around each QTL, since the GWAS resulted in several marker-trait associations with many markers locating at close positions to each other. QTL were first analyzed by interval mapping (IM). The marker with highest LOD values at each putative QTL identified using IM was selected as a cofactor and the selected markers were used as genetic background controls in the approximate multiple QTL model (MQM). The population structure (Q-matrix) was used as covariates. A logarithm of the odds (LOD) threshold value of 3.0 was applied to declare the presence of a QTL at 95% significance level.

### Genomic Analysis of Potential Genes for Salinity Tolerance

The nearest marker of the QTL for salinity tolerance, bPb-9668 on 4H (see results), was consistently detected in all methods. bPb-9668 was located at the end of chromosome 4H. Barley genomic data and gene annotations were downloaded from ftp://ftpmips.helmholtz-muenchen.de/plants/barley/public_data/ (Mayer et al., 2012) and ftp://ftpmips.helmholtz-muenchen.de/plants/barley/public_data/ (Mascher et al., 2013). Annotated genes within 15cM around bPb-9668 on 4H were examined for potential genes for salinity tolerance.

## Results

### Salinity Tolerance of Barley Accessions

**FIGURE 1:**
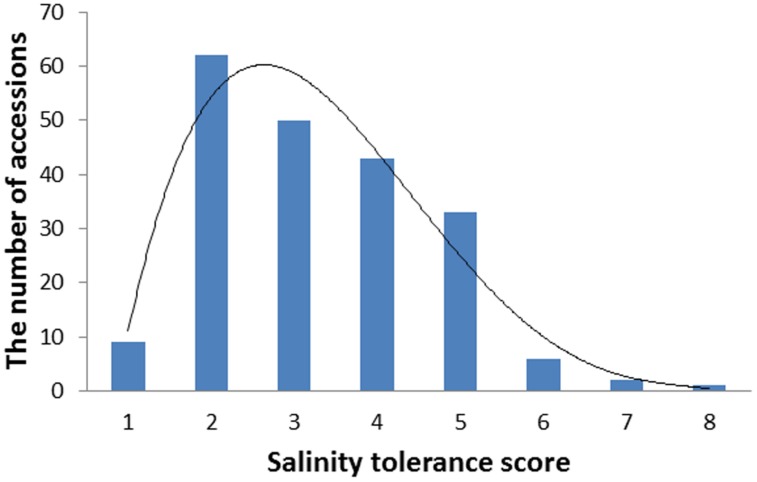
**The distribution frequency of salinity tolerance scores in 206 barley varieties.** Seedlings were treated with 300 mM NaCl at the two leaf stage. Salinity tolerance was scored from 0 to 10 by leaf chlorosis (0 = tolerant, 10 = sensitive). Data were averaged over two growth seasons, 2013 and 2014.

Barley accessions exhibited significant difference in their salinity tolerance. Since the scoring was conducted at a relatively early stage of salt treatment when clear phenotypic segregation was shown, most of the sensitive varieties (e.g., Franklin, Gairdner) were scored for five while the tolerant varieties (CPI-11284-48, TX9425) had a score of 1. The scores from 2 years correlated significantly with each other (*r* = 0.65). Therefore, the average data were used for further analysis. **Figure 1** shows the frequency distribution (the number of accessions) of salinity tolerance based on the average leaf wilting and plant survival scores of all genotypes, ranging from 1 to 8.

### Population Structure

**FIGURE 2:**
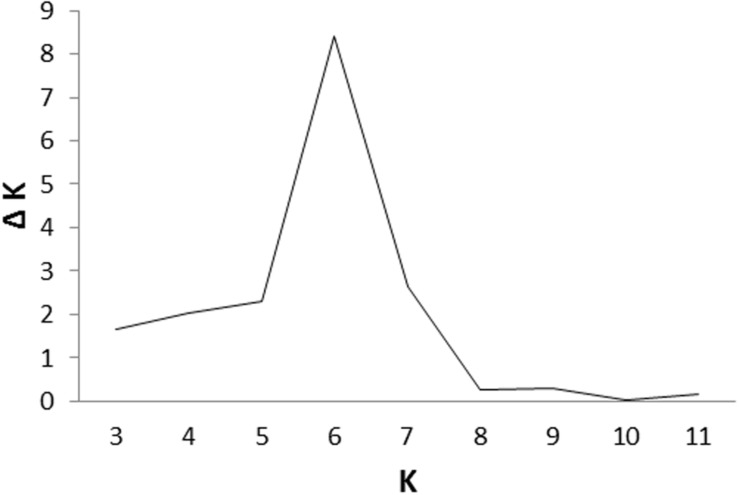
**An estimation of the most probable number of clusters (K), based on 20 independent runs and K ranging from 2 to 12**.

**FIGURE 3:**
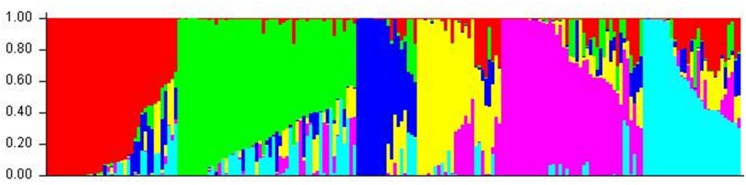
**The population structure of 206 barley accessions**. Six subpopulations (*K* = 6) were produced based on genetic diversity detected by 408 DArT markers, each are represented by a different color.

Cluster parameter K was set from 2 to 12. According to the explanation of Evanno et al. (2005), the largest value of statistic index ΔK was used as an indicator for evaluating the most probable number of subpopulations among all accessions. In this study, ΔK reached the highest value when *K* = 6 (**Figure 2**). Therefore, the most appropriate number of clusters are represented by six different colors (**Figure 3**). STRUCTURE results were also confirmed by PCA (**Supplementary Figure S1**). Details of population structures of 206 barley accessions are listed in Additional File Supplementary Table S1.

### Association Mapping for Salinity Tolerance

**FIGURE 4:**
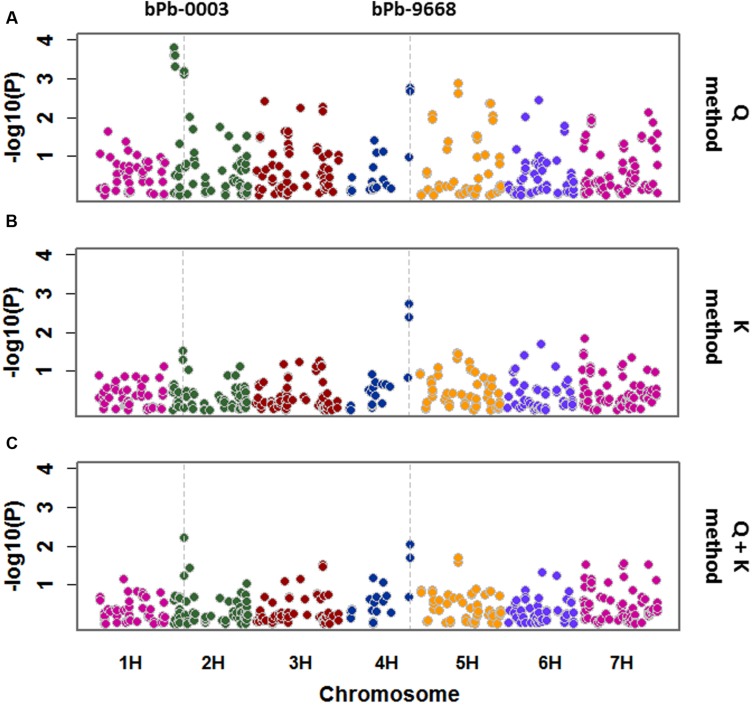
**A Manhattan plot for genome wide association study (GWAS) of salinity tolerance in 206 barley accessions.** GWAS was analyzed by three methods: **(A)** Q method; **(B)** K method; **(C)** Q + K method. Significant associations were identified using criterion of -log_10_ (*P*) > 2 (*P* < 0.01).

Salinity tolerance of 206 barley accessions and 408 DArT markers were used for association mapping. A total of 24 significant marker-trait associations were detected with Q method. These markers are located on 2H, 3H, 4H, 5H, 6H, and 7H (**Figure 4**; **Table 1**), representing 12 potential QTL. Only two significant marker-trait associations (one QTL) were detected on 4H with the K method, while two significant marker-trait associations representing two QTL were identified with the Q + K method, located on 2H and 4H, respectively (**Figure 4**; **Table 1**).

**Table 1:** Association mapping results for salinity tolerance with the Q method, K method, and Q + K method, respectively (*P* < 0.01).

Method	Trait	Chromosome	Position	Marker	*P*	Marker *R*^2^
Q + K (MLM)	SLAV	2H	25.7	bPb-0003	0.0060	0.046
	SLAV	4H	145	bPb-9668	0.0091	0.038
K (MLM)	SLAV	4H	145	bPb-9668	0.0018	0.049
	SLAV	4H	145.1	bPb-5265	0.0041	0.041
Q (GLM)	SLAV	2H	3.5	bPb-5489	0.0002	0.059
	SLAV	2H	3.5	bPb-4285	0.0002	0.056
	SLAV	2H	5	bPb-5191	0.0002	0.056
	SLAV	2H	5.3	bPb-9681	0.0005	0.056
	SLAV	2H	25.7	bPb-8399	0.0006	0.056
	SLAV	2H	25.7	bPb-0003	0.0008	0.049
	SLAV	2H	35.7	bPb-1196	0.0098	0.028
	SLAV	3H	20	bPb-6978	0.0037	0.036
	SLAV	3H	97.4	bPb-6722	0.0055	0.032
	SLAV	3H	145.5	bPb-4156	0.0052	0.033
	SLAV	3H	145.5	bPb-5298	0.0067	0.032
	SLAV	3H	145.5	bPb-5396	0.0068	0.031
	SLAV	4H	145	bPb-9668	0.0017	0.043
	SLAV	4H	145.1	bPb-5265	0.0020	0.040
	SLAV	5H	43.5	bPb-4135	0.0081	0.030
	SLAV	5H	97.9	bPb-2425	0.0024	0.039
	SLAV	5H	98.2	bPb-8101	0.0013	0.044
	SLAV	5H	166.1	bPb-6179	0.0042	0.035
	SLAV	5H	168.3	bPb-0835	0.0042	0.035
	SLAV	5H	168.3	bPb-4595	0.0042	0.035
	SLAV	5H	173.7	bPb-1719	0.0087	0.029
	SLAV	6H	38	bPb-2058	0.0093	0.029
	SLAV	6H	68.2	bPb-5698	0.0034	0.036
	SLAV	7H	140.9	bPb-5923	0.0072	0.031

*^∗^SLAV, Salinity tolerance data are averaged over two growth seasons 2013 and 2014.*

Quantile–quantile (Q–Q) plot was employed to evaluate the fitness and efficiency of different models. The observed –log_10_ (*P*) values for salinity tolerance were closer to expected –log_10_ (*P*) values from the K and Q + K methods than those from the Q method (**Supplementary Figure S2**). However, only one or two QTL were detected in the K or the Q + K methods, whereas about 12 QTL were detected with the Q method.

*P* value < 0.01 have been used as a cut-off for barley GWAS in many studies (Pasam et al., 2012; Shu et al., 2012; Huang et al., 2014). The marker bPb-9668 on 4H (145.0 cM) showed consistent significance (*P* < 0.01) of marker-trait associations using the Q, K, and Q + K methods (**Figure 4**; **Table 1**). Another marker, bPb-5265 (145.1 cM) on 4H which is close to bPb-9668, showed significance under the Q and K methods, not Q + K method (**Table 1**). The marker bPb-0003 on 2H showed significant marker-trait associations with both the Q and the Q + K methods but not the K method (**Figure 4**; **Table 1**).

**FIGURE 5:**
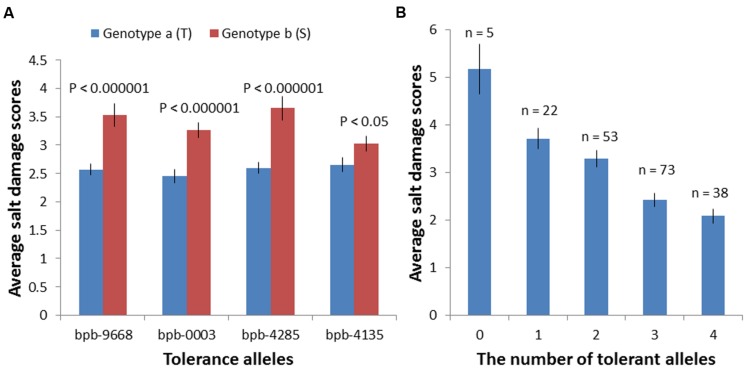
**Salinity tolerance of 206 barley accessions of two genotype groups based on their Base calls of the markers: bPb-9668, bPb-0003, bPb-4285, and bPb-4135. (A)** Accessions with different polymorphisms at these four markers showed very significant differences in salinity tolerance; ^∗^T: tolerant, S: sensitive. **(B)** These four QTL showed an additive effect with the average tolerance score (2.08) of varieties combining all four tolerance alleles than that of varieties with all susceptible alleles (5.167); ^∗^0: without any tolerance alleles, 1–3: with 1–3 tolerance alleles, 4: with all four tolerance alleles.

Based on marker polymorphisms, the salinity tolerance of 206 barley accessions were grouped into two genotypes according to their base calls of the marker bPb-9668 and bPb-0003 (**Figure 5A**; Supplementary Table S2). Accessions with different polymorphisms at bPb-9668 and bPb-0003 showed highly significant differences in salinity tolerance (*P* < 0.0001, **Figure 5A**; Supplementary Table S2).

### QTL Mapping for Salinity Tolerance Using MapQTL6.0 Software

Many marker-trait associations were detected using the Q-method with some of them being located at similar positions. In order to identify the similarity of those markers located at similar positions, MapQTL6.0 was used to detect significant QTL. When analyzed for QTL using MapQTL6.0 software using population structure (Q-matrix) as covariates, the results were very close to association mapping with the Q method (**Supplementary Figure S3**). The analysis produced 3 significant QTL (LOD > 3.0) and 4 tentative QTL (3 > LOD > 2) (**Table 2**), with all of them being in line with those from association mapping with the Q method. The most significant QTL on 4H was the same as that identified with both the K and the Q + K methods (**Tables 1 and 2**). MQM mapping resulted with, apart from two QTL based on bPb-9668 and bPb-0003 from the K or the Q + K method (**Figure 4**; **Table 1**), two more QTL with nearest marker bPb-4285 and bPb-4135 being significant with LOD > 3.0 (**Table 2**). bPb-4285 also showed a highest –log_10_ (P) in the Q method (**Figure 4**; **Table 1**). The salinity tolerance of the 206 barley accessions was also grouped into two genotypes according to the base calls of the marker. As shown in **Figure 5A** and Supplementary Table S2, all four markers showed significant association with salinity tolerance. The four QTL showed additive effects with the average salinity tolerance being increased with the increased number of tolerance alleles. The average damage score of varieties combining all four tolerance alleles was 2.1, while that of no tolerance alleles was 5.2 (**Figure 5B**; Supplementary Table S2).

**Table 2:** QTL mapping results for salinity tolerance in 206 barley varieties when structure was used as covariate (LOD > 2.0).

Trait	Chromosome	Position	Locus	LOD	*R*^2∗^
SLAV^∗∗^	2H	3.5	bPb-4285	3.66	4.8
SLAV	2H	25.7	bPb-0003	2.11	2.6
SLAV	3H	133.5	bPb-6504	2.12	2.7
SLAV	4H	145	bPb-9668	5.67	7.5
SLAV	5H	43.5	bPb-4135	3.91	5.1
SLAV	7H	3.5	bPb-3732	2.13	2.6
SLAV	7H	125.4	bPb-8539	2.47	3.2

*^∗^R^2^: the percentages of phenotypic variation explained by markers.*

^∗∗^SLAV, Salinity tolerance data are averaged over two growth seasons 2013 and 2014.

### Potential Genes for Salinity Tolerance on 4H

In this study, QTL on 4H with the nearest marker of bPb-9668 was the most significant, consistently detected in all methods. Annotated genes around this marker on 4H are listed in Supplementary Table S3. Among all annotated genes, there are two possible genes likely to be associated with salinity tolerance, MLOC_70918.1 and MLOC_5021.1. Both locate at the end of chromosome 4H and close to the marker bPb-9668 according to the POPSeq map (Mascher et al., 2013). MLOC_70918.1 belongs to glutathione-regulated potassium-eﬄux system protein while MLOC_5021.1 is a respiratory burst oxidase-like protein. Ion homeostasis, especially Na^+^ and K^+^, are related to salinity tolerance (Munns and Tester, 2008). *RESPIRATORY BURST OXIDASE HOMOLOG F* (*RBOHF*) encodes a specific isoform of NADPH oxidase, which plays a vital role in soil salinity tolerance (Jiang et al., 2012).

## Discussion

### A New QTL for Salinity Tolerance was Identified by Association Mapping

Salinity tolerance is a genetically and physiologically complex trait controlled by numerous QTL (Flowers, 2004). Leaf wilting and plant survival are two of the major symptoms caused by salt stress and had been used for evaluating salinity tolerance of barley through traditional bi-parental QTL mapping in many studies (Xu et al., 2012; Zhou et al., 2012; Fan et al., 2015). In the present experiments, 206 barley accessions were assessed for salinity tolerance and various mapping methods were used to identify QTL controlling salinity tolerance. Different numbers of QTL were identified using different mapping methods. Association mapping using Q methods identified 12 QTL which are located on 2H (3.5, 25.7 cM), 3H (20, 97.4, 145.5 cM), 4H (145 cM), 5H (43.5, 97.9, 166.1 cM), 6H (38, 68.2 cM), and 7H (140.9 cM), respectively (**Figure 4**; **Table 1**). Most of these QTL were further confirmed by analyzing QTL using MapQTL 6.0 software. Some of them were located at similar positions to those reported before by GWAS or bi-parental QTL mapping. The QTL on 2H located at a similar position to that reported in the DH population of TX9425/Naso Nijo (Xu et al., 2012). QTL on 6H with the nearest marker bPb-2058 was close to QSl.Yy.Fr.6H (26 cM) from the DH population of YYXT and Franklin (Zhou et al., 2012). No QTL was reported for salinity tolerance on 4H at the same position (145 cM) of the QTL identified by all four methods in the current study. The nearest QTL for a salinity tolerance-related trait on 4H was located at 119.1 cM in their consensus map (Close et al., 2009), controlling shoot Na^+^/K^+^ under saline conditions (Long et al., 2013), which is also close to the telomere of 4HL. However, shoot Na^+^/K^+^ under saline conditions was not related to salinity tolerance in their study with the QTL for salinity tolerance being located on 6H (Long et al., 2013).

### GWAS Results Are Affected by Models and Evaluation Methods

In this study, GWAS was conducted with three different models, Q (population structure), K (kinship) and Q+K. According to the Q–Q plots (**Supplementary Figure S2**), K and Q + K were similar, and both stricter than the Q model. The observed –log_10_ (*P*) values for salinity tolerance deviated from the expected –log_10_ (*P*) values in the Q method (general linear model), indicating that they may contain false positive associations (**Supplementary Figure S2A**). The addition of genetic relatedness (i.e., relationship or kinship) makes the MLM more powerful, thus reducing the number of false positive associations (Yu et al., 2006). K and Q + K were similar in this study on the basis of the Q–Q plots and results in **Table 1**, which was in accordance with Cai et al. (2013). However, only two and one QTL were identified with the K method and the Q + K method, respectively, while a lot more QTL were identified with the Q method. Therefore, QTL mapping was also conducted with the MapQTL 6.0 software (Van Ooijen, 2009) using population structure as covariate to adjust the natural variations of this population. Nearly all the QTL identified with MapQTL6.0 were in line with those from association mapping with the Q method and the most significant one was the same as that identified using the K and Q + K methods. The percentages of phenotypic variation explained by various markers analyzed with MapQTL 6.0 are very close to those analyzed with Q method (*R*^2^ = 0.89, **Supplementary Figure S3**).

To compare the robustness of combining different mapping approaches, all the accessions were grouped based on their base calls of the markers bPb-9668, bPb-0003, bPb-4285, and bPb-4135 (**Figure 5**; Supplementary Table S2), the four significant QTL detected with both GWAS (Q method) and MapQTL 6.0 (**Tables 1 and 2**). Accessions with different polymorphisms at bPb-9668, bPb-0003, bPb-4285, and bPb-4135 had differences in tolerance scores of 0.960 (*P* < 0.000001), 0.814 (*P* < 0.000001), 1.053 (*P* < 0.000001), and 0.371 (*P* < 0.05), respectively (Supplementary Table S2). These four QTL also showed additive effects with the average tolerance score (2.1) of varieties combining all four tolerance alleles being significantly better than that of varieties with all susceptible alleles (5.2) (**Figure 5**; Supplementary Table S2). There could be higher chances of false positive or negative errors in GWAS than in bi-parental QTL mapping, resulting from the complex population structure (Myles et al., 2009; Pasam et al., 2012), thus the MLM approach using the K matrix or a combination (Q+K) could perform better than general linkage model (GLM). However, in this study, the K or the Q + K methods were shown to be too strict, resulting in the missing of some possibly useful QTL. QTL mapping using MapQTL6.0 with the Q matrix as covariates in natural populations showed similar power as GLM. The advantage of using the MQM of MapQTL 6.0 is the confirmation of the number of QTL through cofactor selection (Van Ooijen, 2009).

### Confirmation of QTL Identified by GWAS

Salinity tolerance is a quantitative trait controlled by many QTL. Many methods have been used to identify the QTL. Care should be taken to balance the rate of false positives and negatives during the process of analysis using different models/methods (Pasam et al., 2012). Traditional QTL mapping through bi- or multi-parental populations is a powerful method but suffers from a limited amount of recombination. GWAS can partly overcome the limitation by using a diverse germplasm but may lead to a number of false positive or negative associations. Different methods can be complementary to each other and benefit can be achieved by mitigating the other’s limitations (Korte and Farlow, 2013). In this study, the combination of GWAS and QTL mapping has led to successful identification of QTL with potential application in breeding programs. However, the QTL identified by GWAS requires further confirmation in bi- or multi- parental populations.

## Conclusion

In this study, 24 markers showed significant association with salinity tolerance. Different methods were used for QTL detection concluding with four significant QTL. These QTL showed additive effects with salinity tolerance being greatly increased by combining all four tolerance alleles. A new QTL on 4H (telomere of the long arm) was detected with different methods and will be further investigated. Overall, the K or the Q + K method was stricter than the Q method but may result in some missed useful QTL. The Q method, with similar power as MapQTL 6.0 using population structure as covariate, discovered more QTL but could have produced false positives. Population size, accuracy of phenotyping, and quantity of markers can be increased to enhance the power of association mapping, and further confirmation of QTL will be needed. The confirmed QTL can then be used in breeding programs.

## Author Contributions

MZ and CL designed the work; CL and GZ prepared the barley germplasm and did genotyping; MZ and YF did the phenotyping and analyzed the data; SS, Z-HC, and SC contributed to data analysis and interpretation; YF drafted the paper; all authors revised the paper and approved the final version to be published.

## Conflict of Interest Statement

The authors declare that the research was conducted in the absence of any commercial or financial relationships that could be construed as a potential conflict of interest.

## Supplementary Material

FIGURE S1 **Principal component analysis (PCA) of 206 barley accessions.** Population structure and dispersion of the association panel were shown through three dimensional **(A)** and two dimensional **(B)** diagrams. **(C)** Number detection of subpopulations or principal components.

FIGURE S2 **Quantile–quantile (Q–Q) plots of estimated -log_10_ (P).** Q–Q plots were displayed in marker-trait association analysis using three models: **(A)** Q method; **(B)** K method; **(C)** Q + K method. The black line represents the expected line under the null distribution. The red symbol is the observed -log_10_ (P) for salinity tolerance.

FIGURE S3 **Correlations between marker *R*^2^ from the Q method general linkage model (GLM) in GWAS and the *R*^2^ from QTL mapping using MapQTL6.0 software with population structure (Q-matrix) as covariates.** *R*^2^: the percentages of phenotypic variation explained by markers.
