# Supplementary figures and images for: Genome-Wide Association Study Reveals a New QTL for Salinity Tolerance in Barley (Hordeum vulgare L.)
Source: Front Plant Sci. 2016 Jun 28;7:946. doi: 10.3389/fpls.2016.00946 (PMC4923249; doi:10.3389/fpls.2016.00946)

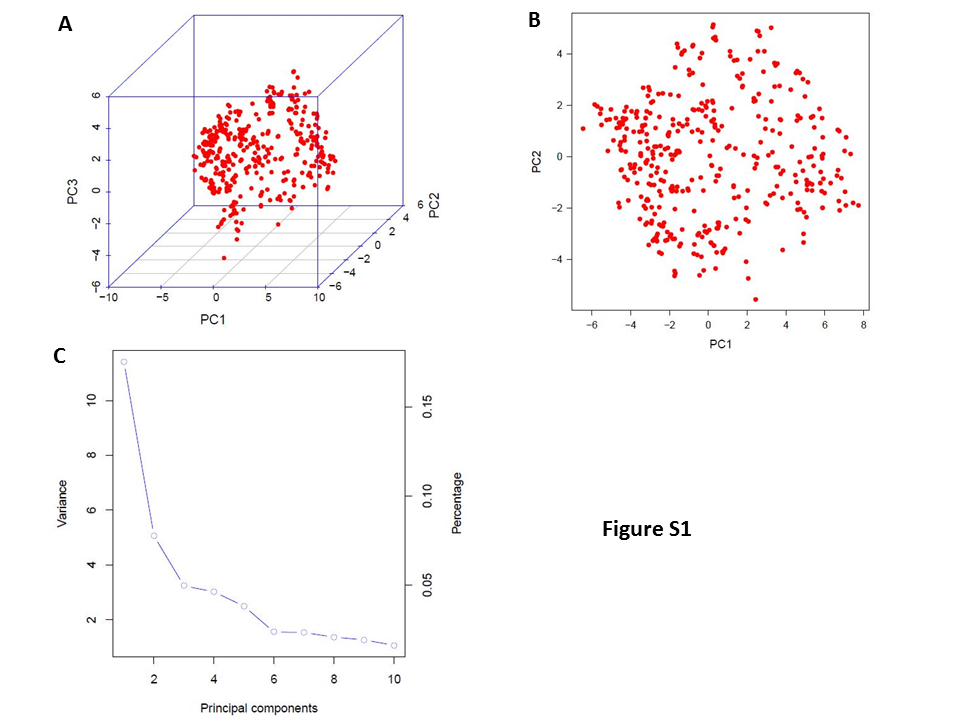

Supplement: FIGURE S1 — Principal component analysis (PCA) of 206 barley accessions. Population structure and dispersion of the association panel were shown through three dimensional (A) and two dimensional (B) diagrams. (C) Number detection of subpopulations or principal components. [file Image_1.TIF]

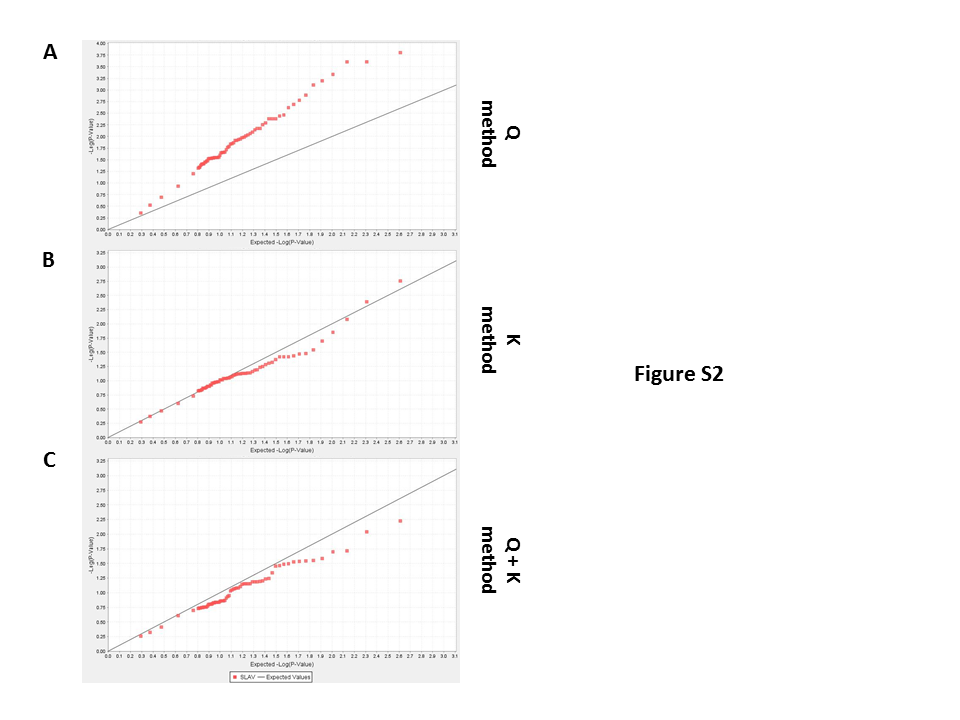

Supplement: FIGURE S2 — Quantile–quantile (Q–Q) plots of estimated -log10 (P). Q–Q plots were displayed in marker-trait association analysis using three models: (A) Q method; (B) K method; (C) Q + K method. The black line represents the expected line under the null distribution. The red symbol is the observed -log10 (P) for salinity tolerance. [file Image_2.TIF]

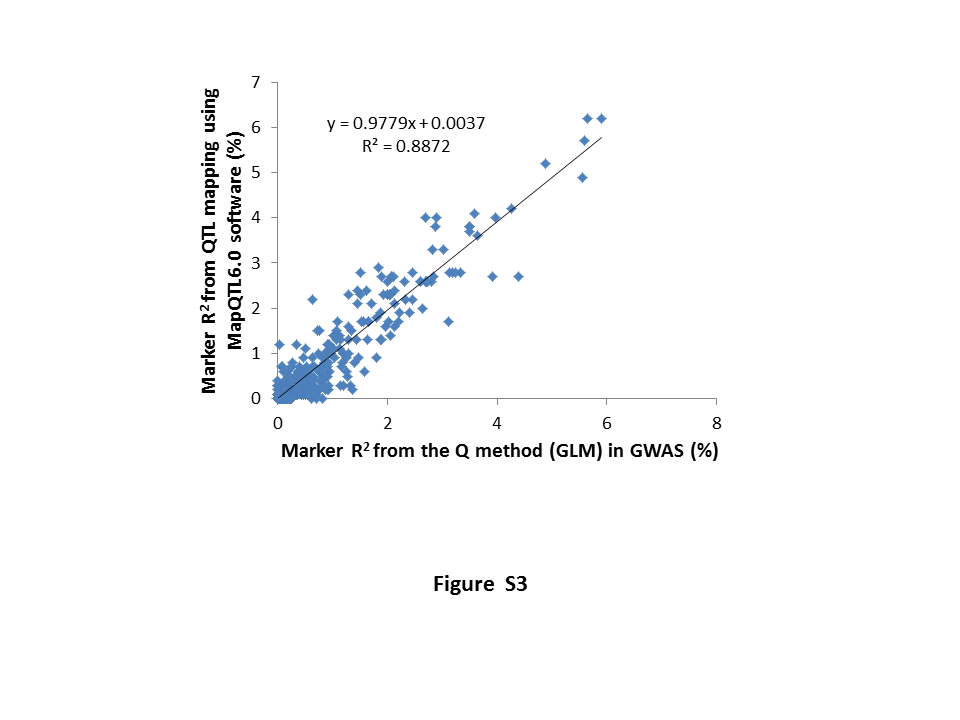

Supplement: FIGURE S3 — Correlations between marker R2 from the Q method general linkage model (GLM) in GWAS and the R2 from QTL mapping using MapQTL6.0 software with population structure (Q-matrix) as covariates. R2: the percentages of phenotypic variation explained by markers. [file Image_3.TIF]
